# Healthcare experiences of pregnant and postnatal women and healthcare professionals when facing child protection in the perinatal period: A systematic review and Critical Interpretative Synthesis

**DOI:** 10.1371/journal.pone.0305738

**Published:** 2024-07-03

**Authors:** Kaat De Backer, Hannah Rayment-Jones, Billie Lever Taylor, Tamsin Bicknell-Morel, Elsa Montgomery, Jane Sandall, Abigail Easter

**Affiliations:** 1 Department of Women and Children’s Health, School of Medicine and Life Course Sciences, King’s College London, London, United Kingdom; 2 Division of Methodologies, Florence Nightingale Faculty of Nursing, Midwifery and Palliative Care, King’s College London, London, United Kingdom; 3 Homerton Healthcare NHS Foundation Trust, London, United Kingdom; Deakin University Faculty of Health, AUSTRALIA

## Abstract

**Background:**

The perinatal period is known as time of transition and anticipation. For women with social risk factors, child protection services may become involved during the perinatal period and this might complicate their interactions with healthcare providers.

**Aim:**

To systematically review and synthesise the existing qualitative evidence of healthcare experiences of women and healthcare professionals during the perinatal period while facing child protection involvement.

**Methods:**

A systematic search of databases (Web of Science, MEDLINE, EMBASE, PsychINFO, CINAHL, ASSIA, MIDIRS, Social Policy and Practice and Global Health) was carried out in January 2023, and updated in February 2024. Quality of studies was assessed using the Critical Appraisal Skills Programme. A Critical Interpretative Synthesis was used alongside the PRISMA reporting guideline.

**Results:**

A total of 41 studies were included in this qualitative evidence synthesis. We identified three types of healthcare interactions: Relational care, Surveillance and Avoidance. Healthcare interactions can fluctuate between these types, and elements of different types can coexist simultaneously, indicating the complexity and reciprocal nature of healthcare interactions during the perinatal period when child protection processes are at play.

**Conclusions:**

Our findings provide a novel interpretation of the reciprocal interactions in healthcare encounters when child protection agencies are involved. Trust and transparency are key to facilitate relational care. Secure and appropriate information-sharing between agencies and professionals is required to strengthen healthcare systems. Healthcare professionals should have access to relevant training and supervision in order to confidently yet sensitively safeguard women and babies, while upholding principles of trauma-informed care. In addition, systemic racism in child protection processes exacerbate healthcare inequalities and has to be urgently addressed. Providing a clear framework of mutual expectations between families and healthcare professionals can increase engagement, trust and accountability and advance equity.

## Introduction

Pregnancy and the postnatal period are times of transition and anticipation. They can also be times during which women have more contact with healthcare services than at any other given time period in their life [1]. Universally, a schedule of routine antenatal and postnatal appointments is put in place, to monitor the physical and emotional wellbeing of mother and baby [2]. For women with medical or socio-economic vulnerabilities, pregnancy is often referred to as a ‘window of opportunity’, whereby the anticipated arrival of a newborn baby acts as a newly found incentive to address ongoing health and social risk factors [3–6]. Regular contact with healthcare professionals (HCPs) can curtail previous existing barriers to disclosure, detection and treatment and can facilitate referrals to appropriate support services, such as smoking cessation, mental health support, and drug and alcohol services [2–5, 7].

Some families will require support to meet the physical, developmental or emotional needs of their babies, which can be provided by child protection (CP) agencies. In the most concerning circumstances, mandatory CP processes can be instigated during pregnancy when risks are identified endangering the wellbeing and safety of the unborn baby [8]. A referral to CP agencies is often initiated by HCPs involved in healthcare during the perinatal period, such as midwives in maternity services. While it is universally accepted that HCPs have a duty of care towards the (unborn) baby and must report any safeguarding concerns, it is equally accepted that this aspect of their professional role complicates the ongoing relationship with parents(-to-be) [9, 10]. Engagement with healthcare services is crucial to ensure the health of mother and baby, whilst also contributing information to CP agencies when safeguarding assessments are being carried out [8, 11, 12]. For some, fear of their baby being taken into State Care can leave women reluctant to access antenatal services and share their personal circumstances, leaving HCPs being unaware of their wider psychosocial support needs [7, 13, 14]. Previous negative experiences with HCPs can further complicate these interactions [15, 16]. For others, fear of potential consequences can act as a motivating factor to work co-operatively with HCPs, in order to maximise their opportunity to evidence change and alleviate professional concern [7]. Generally, women who engage with professionals during pregnancy, have described the sudden involvement of a variety of agencies as uncoordinated and overwhelming [6]. Healthcare encounters during the perinatal period are therefore complex, reciprocal interactions between those seeking healthcare and those providing it (HCPs). These relationships become even further compromised when CP agencies proceed to request separation of the infant after birth, due to the severity of safeguarding concerns. In the UK, the legal decision to separate a newborn from its birth mother, in some cases as soon as a few hours after birth, is embedded in the Children’s Act 1989 and is in principle a temporary safeguarding intervention with the possibility of reunification [17]. While rates of infants (under the age of one) being placed in State Care nearly doubled over the last decade, reaching 5,540 in 2023 [18], reunification rates are low, accounting for about one in five newborns being returned home to the care of their mother [19]. This means the decision to separate an infant from their mother is often perceived with a sense of permanency, by both professionals and birth parents. A literature review by Mason et al. on infant removal at birth described the psychological impact on birth mothers as acutely traumatic, leaving women in an heightened state of vulnerability, marked by intense feelings of loss, grief and shame [12]. Conjointly, separation at birth has been described as the most distressing and harrowing aspect of contemporary midwifery practice [9, 13, 20].

Mother-infant separation due to CP processes has been associated with increased maternal morbidity and mortality. A Canadian study by Wall-Wieler et al. (2018) looked at mortality rates of women who had a child taken into care, compared to those that did not. They defined avoidable causes of death as those cases where death could have been prevented or treated, such as infections, certain cancers and intentional or unintentional injuries [21]. The study used a cohort of biological sisters who were both mothers, whereby one had a child taken into care (Group 1, n = 1,974) and the other did not (Group 2, n = 1,974). The study found that—after adjusting for individual differences and family characteristics—women in Group 1 were 3.23 times more likely to die than those in Group 2, with greater risks for avoidable causes (adjusted HR = 3.46) than for unavoidable causes (HR = 2.92). Even when comparing with mothers who experienced the death of a child, mothers in Group 1 were still 2.71 times more likely to die from avoidable causes [21]. Similar evidence for both mothers and fathers has been found in a Swedish national cohort study, which highlighted an increased risk of suicide among mothers, as well as ischaemic heart disease [22]. In the immediate postnatal period, women whose baby is taken into care face an acute psycho-social crisis, which can lead to a return to harmful coping strategies, such as a relapse of drugs and alcohol use [23, 24]. Mothers who are separated from their baby within a week after birth have higher odds ratios of experiencing postnatal depression compared to women with lower levels of child protection involvement (AOR = 1.80; 95% CI 1.43, 2.17) or those without (AOR = 2.46; 95% CI 1.80, 3.36) [25]. In the UK, one in four women will go through the same legal process of infant removal within 10 years of the first removal, often after the birth of another baby, with the highest risk within the first three years of the initial proceedings [26]. This sequence of rapid repeat pregnancies carries significant health risks for both mothers and babies, such as preterm birth and low birth weight [27, 28] and compounds previous trauma and loss [26].

Previous descriptive reviews by Simkiss (2013) and Wilkinson and Bowyer (2017) on risk factors associated with children entering State Care have identified a range of maternal factors, such as low socio-economic status, single parenthood, ethnicity, age, disability, smoking in pregnancy, mental illness, learning disability, adverse childhood experiences, parental history of crime, and intergenerational cycles of child maltreatment [7, 29, 30]. Pre-existing social risk factors and maternal vulnerabilities, as well as increased risks of post-separation maternal morbidity and mortality require comprehensive and multi-disciplinary care pathways to meet women’s physical and emotional needs. However, it has been argued that women with CP involvement are often seen as less deserving of high-quality healthcare or have less ‘candidacy’. Dixon-Woods (2006) used the term ‘candidacy’ to describe how people’s eligibility for care is negotiated between themselves and healthcare professionals and services [31]. In addition, it is reported they encounter more paternalistic care, often perceived as surveillance [32]. A recent meta-ethnography by Heys et al. (2021) found that disadvantaged and vulnerable women had overwhelmingly negative experiences of maternity care, which left them disillusioned, with feelings of stigma and shame [33]. Yet, key features of good practice care models to support parents in the context of child protection processes have been described by Grant et al. (2023): such interventions require relationship-based, trauma-informed, multidisciplinary and long-term approaches to meet parental complex health needs [34]. However, the discrepancy between best practice care models for vulnerable women and families involved in CP processes and the lived reality of current care pathways requires further research. Grant et al.’s review highlighted the need for further research into the role of maternity services to support complex health needs when facing separation. Our review aims to fill this evidence gap, by looking at healthcare interactions across healthcare services involved during the perinatal period.

Therefore, the aim of this review was to systematically gather, appraise, synthesise and interpret the existing qualitative evidence of experiences of healthcare during the perinatal period while facing CP processes. Our review aims to bring together the experiences of pregnant and postnatal women who access healthcare in the perinatal period when facing child protection processes, as well as the experiences of HCPs providing care to these women. Findings were synthesised and interpreted to generate a theoretical framework of healthcare interactions to inform clinical care.

## Methods

### Research design

We undertook a systematic literature search of the existing qualitative evidence and a Critical Interpretative Synthesis informed by Dixon-Woods [31]. Similar to Noblit and Hare’s principles of meta-ethnography [35], Critical Interpretative Synthesis aims to go further than describing (or aggregating) what has been previously reported [31, 35]. In our case, we anticipated that included studies would span a wide range of healthcare settings, and we aimed to go beyond data synthesis to create a new conceptual framework of healthcare interactions amidst CP processes. Critical Interpretative Synthesis, with a focus on theory-generation, was found to be best suited to meet these objectives whilst providing more flexibility than Noblit and Hare’s Meta-ethnography approach [31, 36, 37].

### Review team reflexivity

The review team consisted of academics and clinical academics, with backgrounds in midwifery (KDB, HRJ, TB, EM, JS) sociology (JS) and psychology (BLT, AE). Several team members have extensive clinical experience of providing enhanced midwifery care to women in vulnerable positions, warranting social services involvement, whether this is due to substance use disorders (TB), severe or complex mental health needs and learning disabilities (KDB), or a range of social risk factors (HRJ). One team member (BLT) is a clinical academic with experience of working in perinatal mental health services and in Looked After children services (i.e. where a child has been removed from parental custody). As such, the team has first-hand professional experience of working in a multi-disciplinary context of healthcare, alongside children’s social care and other agencies and third sector involvement.

### Lived experience involvement

This review was undertaken in the context of a doctoral research study (MUMS@RISC study) funded by the National Institute of Health and Care Research (NIHR). A designated advisory panel of women with lived experience of infant removal shortly after birth was formed for the purpose of continuous lived experience involvement throughout the entire project. Principles of trauma-informed patient and public involvement [38] have been at the core of the advisory panel, with the co-created MUMS@RISC Charter for Research Engagement. The panel is consulted at regular intervals throughout the doctoral study and initial findings of this review were discussed during panel meetings in November 2023 and January 2024, and informed the development of the final theoretical framework and synthesising concepts. A first draft of the visual display of review findings was discussed and co-designed and the panel’s feedback was incorporated in the final version. Members of the MUMS@RISC advisory panel are reimbursed for their time, with regular check-in provided as part of the wider doctoral study lived experience involvement work.

### The review question and search strategy

We followed PRISMA reporting guidelines [39] (S1 Table) and prospectively registered the study protocol on PROSPERO (The International Prospective Register of Systematic Reviews, registration number CRD:42022381632). The review question was informed by the PerSPE©TIF framework, which has been reported to be well-suited for qualitative evidence syntheses [40] and defined as follows: *“What are the experiences of pregnant and postnatal women and healthcare professionals regarding healthcare while facing child protection in the perinatal period*?*”*

The search strategy, with its pre-defined inclusion and exclusion criteria as outlined in Table 1, was carefully designed in consultation with the review team and with valuable support from a university librarian and followed the same PerSPE©TIF framework. Aspects of the search strategy also built on previous work by one member of the review team (BLT) [41]. Various draft versions of the search strategy were piloted through scoping exercises in different databases, before the formal run of the database searches. The search strategy included MeSH headings and free-text terms based on the key concepts of the PerSPE©TIF framework, with subject headings adapted in line with the particular database and can be found in S1 Table. For this review a broad definition of healthcare as the ‘phenomenon of interest’ was used, meaning any intervention, contact or effort to prevent, detect, diagnose, treat, improve or cure physical and mental disease or impairment, provided by trained healthcare professionals. This included both hospital-based and community care, including home visits.

**Table 1:** Inclusion and exclusion criteria.

	Inclusion criteria	Exclusion criteria
1. Population	Study focussing on perspectives of: ◾ pregnant women accessing heath care **OR** ◾ Studies focussing on perspectives of recent mothers (birth in the last year) accessing healthcare **OR** ◾ Healthcare professionals working in a healthcare setting who provide care to pregnant or postnatal women. This includes maternity care, mental health care, drug and alcohol services, etc….	Studies focussing on perspectives of: Mothers with older children (>1y) **OR** ◾ Non-pregnant women **OR** ◾ Other family members, including fathers **OR** ◾ Social workers or professionals working in statutory social services and agencies, and not providing care in a healthcare or therapeutic setting
2. Phenomenon of interest	For women: Experiences of receiving (health)care	◾ Studies solely focussing on the child protection process or court process ◾ Studies focussing on experiences of motherhood in context of child protection, without mentioning healthcare
	For professionals: Experiences of providing (health)care	
3. Environment	Studies that put the healthcare experience in a context of: Child removal ◾ Voluntary or involuntary loss of custody ◾ Child protection processes	Studies where this context is not mentioned
4. Timeframe	Studies that focus on healthcare during pregnancy and / or the first year after birth	Studies with a focus on healthcare outside the perinatal period
5. Studies	◾ Qualitative studies or qualitative components from mixed methods studies including (but not limited to) designs such as phenomenology, grounded theory, ethnography, case study, narrative research, action research, feminist research and qualitative description; ◾ Qualitative studies or components from mixed methods studies in which data is collected directly from participants or obtained through direct observation of participants.	◾ Reviews, editorials, dissertation abstracts or any other paper that does not present primary data of an empirical study; ◾ Studies that only report quantitative data; ◾ Theoretical and methodological articles, systematic reviews, meta-analysis, and commentaries.

Eligible studies in English were identified using database specific search strategies in nine electronic databases: Web of Science, MEDLINE, EMBASE, PsychINFO, CINAHL, Maternity and Infant Care (MIDIRS), Social Policy and Practice, Applied Social Sciences Index and Abstracts (ASSIA) and Global Health. Database searches were conducted between January 4^th^ and 30^th^ 2023 and updated on February 16^th^ 2024, to ensure we captured any more recent literature. We carried out forward and backward citation tracking as well as reference list screening of included studies to identify additional relevant primary research studies, and used OpenGrey and ProQuest to search for relevant unpublished primary research.

### Search outcome and screening

Searches were carried out by the first author, with search results uploaded in Covidence software, and duplicates removed. All titles and abstracts were double-screened, with one reviewer (KDB) screening all search results and members of the review team (AE, HRJ, TB, EM and BLT) conducting the second screen. Full text screening followed a similar double-screening approach. Discrepancies were resolved through discussion and consultation during regular review meetings with the wider review team and a decision log was available to the entire review team, to document the decision-making process.

### Quality appraisal

The quality of included studies was appraised using the Critical Appraisal Skills Programme (CASP) qualitative checklist [42], which is the most commonly used appraisal tool for qualitative studies and endorsed by the Cochrane Qualitative and Implementation Methods Group [43]. The checklist consists of three sections with a total of ten questions to assess the methodological quality and rigour of included studies: the first two sections (A: ‘Are the results of the study valid?’ and B: ‘What are the results?’) contain nine questions with yes/can’t tell/no answers. In addition to the methodological quality and rigour of studies, we also identified service-user involvement and trauma-informed approaches as additional areas of assessment relevant to our review. The review team therefore included the following questions to assess principles of co-design, public and patient involvement and trauma-informed approaches in each of the included studies: 1) Was there a trauma-informed approach or distress protocol for participants in place?; 2) Does the study report on any patient and public involvement activities as part of the study design? We categorised studies for both questions as ‘none reported’ if there was no mentioning at all of any relevant items, ‘some reported’ if studies mentioned at least one relevant element (e.g. consideration of a safe environment, check-in, participant reimbursement, piloting interview guide, etc.), and ‘considered’ if studies reported multiple elements of the above. All studies were rated against the nine CASP questions and the additional questions as described above by the first author, with a subset of 20% of included studies doubly rated by a member of the review team. No disagreements on quality ratings occurred. The overall quality of each study was categorised as ‘strong’ (CASP score 7–9), ‘moderate’ (CASP score 4–6) or ‘weak’ (CASP score 0–3). Only studies considered ‘strong’ or ‘adequate’ were included to support trustworthiness and credibility of the review findings. No studies were excluded on the basis of lack of public and patient involvement or trauma-informed approaches, but findings were interpreted with careful consideration of the ethical shortcomings of these studies.

### Data extraction

A moderate selective approach to data extraction was adopted in order to only extract findings substantiated by direct data or quotations and relevant to the research question [44]. A designated data extraction form was created in Covidence software to facilitate data extraction and the first author extracted all relevant data accordingly. Data extraction of a subset of 20% of included studies was checked by members of the review team and consensus was reached where this was required. All extracted data was imported into NVivo software under a unique study ID for each included study.

### Data synthesis and assessment of confidence in the review findings

Data were synthesised according to Dixon-Wood’s principles of Critical Interpretative Synthesis (CIS) [31] and guided by several worked examples in the field of midwifery and nursing [36, 45–47]. CIS has two distinct components that transcend a descriptive or aggregative approach to evidence synthesis: Phase 1) initial exploration and inspection of the included studies similar to that undertaken in primary qualitative research, with identifying recurrent themes and developing a critique of the existing evidence; Phase 2) generating a synthesising argument [31]. The first phase of data synthesis as part of CIS was carried out with NVivo software, iteratively generating an inductive and data-driven codebook (S3 Table). We initially coded studies focussing on women’s experiences and subsequently focussing on studies capturing HCPs’ experiences. We then returned to the women’s papers to sense-check the single coding frame generated.

During the second phase, the different codes were grouped together to facilitate the next step of data synthesis, i.e. the creation of a ‘coherent theoretical framework’, before reaching the final stage of the synthesis, i.e. the development of a ‘synthesising construct’ [31]. This work was led by the first author and refined through iterative feedback with the wider review team and sense-checked by the members of the MUMS@RISC advisory panel. Their feedback was incorporated in the final study results as presented in this paper.

### Search results

**Fig 1:**
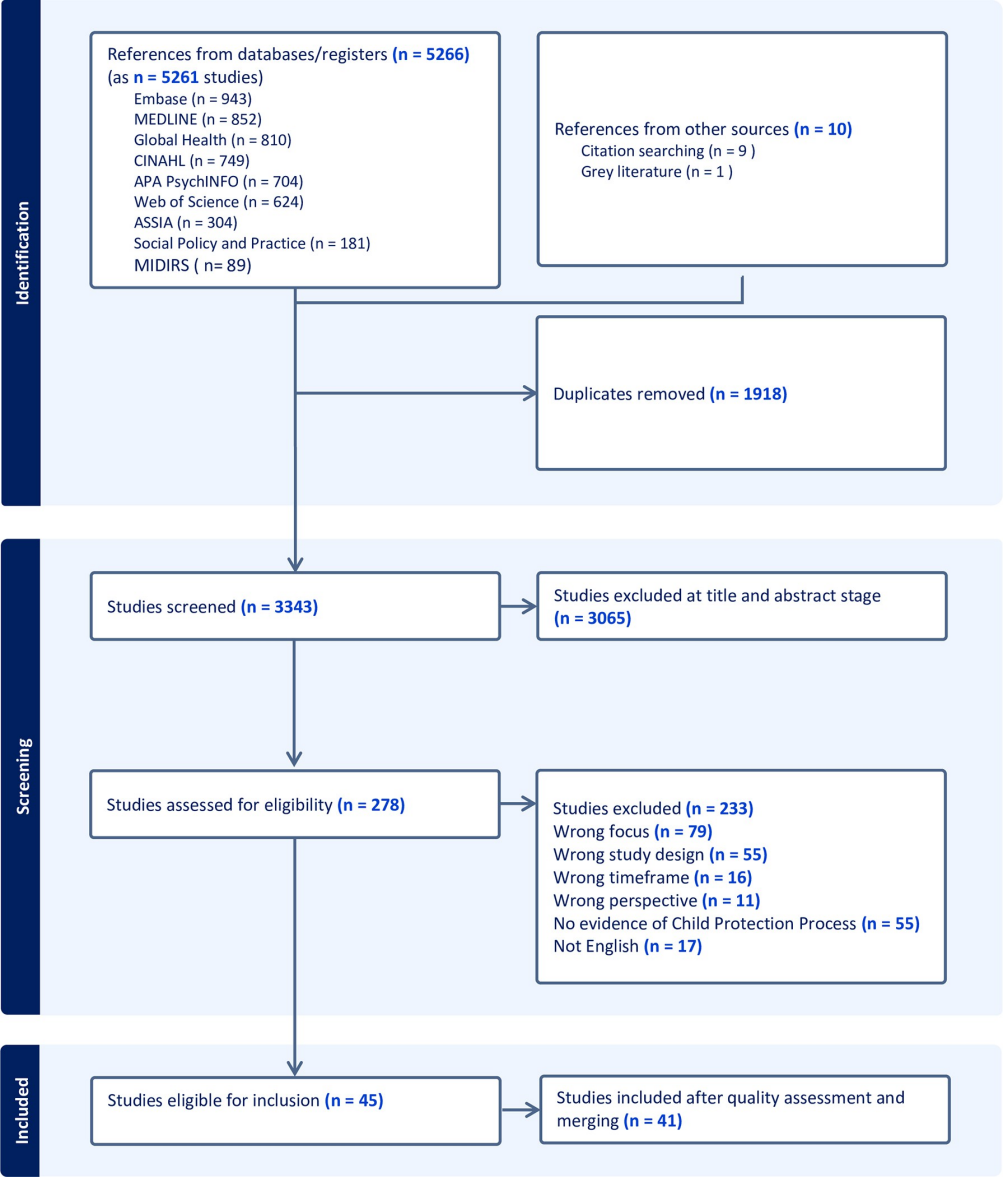
PRISMA diagram.

Overall, 5,261 search results were retrieved from database searches, grey literature and citation tracking and imported in Covidence. A total of 3,343 studies were screened for title and abstracts after duplicates were removed (n = 1,918). The full text of 278 studies were assessed for eligibility, with 46 studies meeting all inclusion criteria. The PRISMA diagram is presented in Fig 1. Subsequently one study was excluded due to overall poor methodological quality [48] and four studies were merged at a later time as they were found to report on the same study [9, 49–51].

### Description of included studies

A total of 41 studies were included in this review, including three PhD dissertations and five studies with findings published over two separate peer-reviewed papers, all of which were included in the review. A full overview of characteristics and quality assessments of included studies can be found in Table 2.

**Table 2:** Description of included studies.

First Author & Title	Perspectives	Participants	Setting	Data collection and Qualitative Analysis Methodology	Quality Assessment		
					Criteria 1*	Criteria 2*	Criteria 3***
**1. Aston et al. (2021)** Mothers’ Experiences with Child Protection Services: Using Qualitative Feminist Poststructuralism	Women’s perspectives	N = 5 first time mothers	Family resource centre Nova Scotia, Canada	Semi-structured focus groups Feminist poststructuralism (Discourse Analysis)	Strong	Considered	None reported
**2. Ayerle et al. (2011)** Key role in the prevention of child neglect and abuse in Germany: Continuous care by qualified family midwives	Women’s perspectives	N = 14 mothers who received midwifery care from "Family midwives"	Family midwives Lower Saxony, Germany	Problem-focused interviews Single case analysis; Comprehensive data analysis	Strong	Some	None reported
**3. Barbosa et al. (2022)** Attention to Women’s Sexual and Reproductive Health at the Street Outreach Office	Healthcare professionals’ perspectives	N = 9 staff members of a Street Outreach Office	Street Outreach Office Brazil	Qualitative semi-structured interviews Thematic content analysis	Strong	Some	None reported
**4. Burduli et al. (2022)** Supporting perinatal individuals with opioid use disorder and their newborns experiencing neonatal abstinence syndrome: impressions from patients and healthcare providers	Women and healthcare professionals’ perspectives	N = 10 pregnant women in Opioid Addiction Treatment or postpartum; N = 10 healthcare providers	Opioid Antagonist Therapy programs; perinatal healthcare providers and local hospitals (NAS providers) Washington, United States of America	Semi-structured interviews Descriptive qualitative content analysis	Adequate	Some	None reported
**5. Castell et al. (2016)** Midwives’ experiences of caring for women with learning disabilities: A qualitative study	Healthcare professionals’ perspectives	N = 9 midwives	Maternity service United Kingdom	Semi-Structured interviews Interpretive Phenomenological Analysis	Strong	Some	Some
**6. Chandler et al. (2013)** Substance, structure and stigma: Parents in the UK accounting for opioid substitution therapy during the antenatal and postnatal periods	Women’s perspectives	N = 45 parents with drug addiction	NHS services South East Scotland, United Kingdom,	Qualitative, longitudinal semi-structured interviews over three timepoints Narrative approach to thematic coding	Adequate	Some	None reported
**7. Coupland et al. (2021)** Developing a model of care for substance use in pregnancy and parenting services, Sydney, Australia: Service provider perspectives	Healthcare professionals’ perspectives	N = 38 staff members in SUPPS	Substance Use in Pregnancy and Parenting Services (SUPPS) Sydney, Australia	Semi-structured interviews Grounded Theory Approach	Strong	None reported	Considered
**8. Crawford et al. (2022)** Stigmatization of pregnant individuals with Opioid Use Disorder	Women’s perspectives	N = 23 women with opioid use relapse or near-miss overdose during the perinatal period	Addiction treatment providers Texas, United States of America	Qualitative interviews Content analysis using Reproductive Justice Framework	Strong	Considered	None reported
**9. Everitt et al. (2015 & 2017) (2 papers)** Midwives’ experiences of removal of a newborn baby in New South Wales, Australia: Being in the “head” and “heart” space Working with Vulnerable Pregnant Women Who are at Risk of Having their Baby removed by the Child Protection Agency in New South Wales, Australia	Healthcare professionals’ perspectives	N = 10 midwives	Maternity services New South Wales, Australia	In-depth interviews Thematic analysis	Strong	None reported	None reported
**10. Frazer et al. (2019)** Treatment for substance use disorder in pregnant women: Motivations and barriers	Women’s perspectives	N = 20 pregnant women in substance use disorder treatment	Center for Addiction and Pregnancy (CAP) Baltimore, United States of America	Directed interviews Thematic analysis	Adequate	None reported	None reported
**11. Gilchrist et al. (2012)** Reducing depression among perinatal drug users—what is needed? A triangulated study	Women and healthcare professionals’ perspectives	N = 15 pregnant women postnatal mothers attending a treatment centre for addiction; N = 13 care providers N = 10 ’experts’	Women’s hospital Melbourne, Australia	Sequential exploratory design, using semi-structured interviews Thematic analysis and content analysis; Grounded theory	Adequate	Some	None reported
**12. Gordon et al. (2019)** Influence of past trauma and health interactions on homeless women’s views of perinatal care: a qualitative study	Women’s perspectives	N = 11 women who experienced homelessness during pregnancy	Three community settings United Kingdom	Semi-structured interviews Iterative thematic data analysis	Strong	Some	Some
**13. Harvey et al. (2015)** Hope amidst judgement: the meaning mothers accessing opioid treatment programmes ascribe to interactions with health services in the perinatal period	Women’s perspectives	N = 6 women under care of an opioid treatment clinic during the perinatal period	Drug and Alcohol service (Opioid treatment clinic) Sidney, Australia	Narrative inquiry through interviews (two timepoints) Narrative analysis	Strong	Considered	None reported
**14. Herriott (2019 & 2024) (PhD dissertation and paper)** Prenatal Care for Women with Substance Use Disorders: Perspectives of Women and Health Care Providers “I just want the best for him.” Pregnancy in the context of substance use disorders: Perspectives of postpartum women	Women and healthcare professionals’ perspectives	N = 19 women who gave birth to a newborn exposed to opiates or illicit substances; N = 10 healthcare professionals (PhD only)	New Hampshire, Massachusetts, Rhode Island, United States of America	In-depth semi-structured interviews Theory-driven and data-driven Thematic Analysis	Strong	Considered	None reported
**15. Hughes et al. (2024)** Antenatal care of women who use opioids: a qualitative study of practitioners’ perceptions of strengths and challenges of current service provision in Scotland	Healthcare professionals’ perspectives	N = 13 health and social care professionals providing perinatal care to women using opioids	Scotland	Semi-structured interviews Framework Analysis	Strong	None reported	None reported
**16. Jarlenski et al. (2019)** Obstetric and Pediatric Provider Perspectives on Mandatory Reporting of Prenatal Substance Use	Healthcare professionals’ perspectives	N = 20 healthcare professionals providing care to substance-using pregnant women	Obstetric and paediatric clinical care settings Pennsylvania, United States of America	Individual interviews Content Analysis	Strong	None reported	Some
**17. Jessup et al. (2003)** Extrinsic barriers to substance abuse treatment among pregnant drug dependent women	Women’s perspectives	N = 36 pregnant or postnatal women in residential treatment of alcohol or drugs dependency	Substance abuse treatment programs for pregnant and parenting women Northern California, United States of America	Semi-structured life history interviews Life history analysis	Strong	Some	None reported
**18. Kearney (1993) (PhD dissertation)** Salvaging self: A grounded theory study of pregnancy on crack cocaine	Women’s perspectives	N = 40 pregnant women and N = 20 postpartum women using drugs during current or most recent pregnancy	West Coast, United States of America	Semi-structured interviews Cross-sectional Grounded theory analysis	Strong	Considered	Considered
**19. Khan et al. (2021)** A Socio-Ecological Approach to Understanding the Perinatal Care Experiences of People with Intellectual and/or Developmental Disabilities in Ontario, Canada	Women’s perspectives	N = 10 women with intellectual and/or developmental disability	Ontario, Canada	Semi-structured interviews Content analysis approach	Strong	Considered	Considered
**20. Lamb et al. (2008)** Exploring experiences and attitudes about health care complaints among pregnant women, mothers and staff at an Opioid Treatment Service	Women and healthcare professionals’ perspectives	N = 13 women; N = 10 healthcare staff	Opioid Treatment Service and associated Child and Maternity Services in hospital settings New South Wales, Australia	Semi-structured interviews Thematic analysis	Strong	Some	None reported
**21. Marsh, C.A. et al. (2019)** Making the hidden seen: A narrative analysis of the experiences of Assumption of Care at birth	Women and healthcare professionals’ perspectives	N = 3 mothers; N = 7 midwives	New South Wales, Australia	Individual interviews Narrative inquiry	Strong	Considered	Some
**22. Marsh, W et al. (2020) (2 papers)** Babies removed at birth: mothers’ and midwives’ narratives Removal of babies at birth and the moral distress of midwives	Women and healthcare professionals’ perspectives	N = 4 mothers; N = 8 midwives	England	Narrative inquiry: interviews using photo-elicitation techniques (mothers) Focus groups with midwives, incorporating photo-elicitation techniques;LEARNS methodology	Adequate	None reported	Some
**23. Mitchell-Foster et al. (2022)** Disconnected perspectives: Patient and care provider’s experiences of substance use in pregnancy	Healthcare professionals’ perspectives	N = 15 healthcare providers	Regional hospital British Columbia, Canada	Patient journey mapping; Semi-structured interviews Inductive thematic coding	Strong	None reported	Some
**24. Morris et al. (2012)** Drugs and having babies: An exploration of how a specialist clinic meets the needs of chemically dependent pregnant women	Women’s perspectives	N = 20 pregnant women	A specialist antenatal clinic for chemically dependent pregnant women Melbourne, Australia	Critical ethnography Narrative inquiry	Strong	None reported	Considered
**25. Morrison et al. (2023)** Barriers to care for pregnant and post-partum women experiencing co-occurring intimate partner violence and opioid use disorder	Healthcare professionals’ perspectives	N = 49 service providers	A range of context where care is provided to women experiencing intimate partner violence and opioid use disorder, United States of America	Semi-structured interviews Thematic analysis	Strong	Some	None reported
**26. O’Connor et al. (2020)** The experiences of pregnant women attending a specialist service and using methamphetamine	Women’s perspectives	N = 20 pregnant women attending a specialist Drug and Alcohol service	A Women and Newborn Drug and Alcohol Service (WANDAS) Perth, Western Australia	Semi-structured life interviews Hermeneutic phenomenology	Strong	Considered	None reported
**27. Olaniyan (2021) (PhD Dissertation)** Implicit racial bias in prenatal visit patient-clinician communication, prenatal screening, and intervention	Women’s perspectives	N = 479 pregnant patients and their clinician at first obstetric visit	Five outpatient obstetric clinics, United States of America	Observational data from 479 first obstetric visit; semi-structured interviews (N = 85) Thematic Analysis	Strong	Some	None reported
**28. Oni et al. (2022)** Barriers to women’ s disclosure of and treatment for substance use during pregnancy: A qualitative study	Women’s perspectives	N = 15 women with a history of substance use during pregnancy	Melbourne, Australia	Semi-structured interviews Thematic analysis	Strong	None reported	None reported
**29. O’Rourke-Schoff et al. (2020)** The labour and birth experience of women with opioid use disorder: A qualitative study	Women’s perspectives	N = 9 mothers with opioid use disorder and a history of sexual violence	Massachusetts, United States of America	Semi-structured interviews Inductive content analysis	Strong	Some	None reported
**30. Premkumar et al. (2019)** “A Resume for the Baby”: Biosocial Precarity and Care of Substance-Using, Pregnant Women in San Francisco	Women’s perspectives	N = 15 women with perinatal illicit drug abuse (paper is case study of one pregnant woman)	General hospital setting San Francisco, United States of America	Semi-structured interviews Grounded theory approach	Adequate	Some	None reported
**31. Proulx et al. (2020)** The Lived Experience of Postpartum Women Attending Outpatient Substance Treatment for Opioid or Heroin Use	Women’s perspectives	N = 10 mothers enrolled in outpatient substance use treatment program	Outpatient substance treatment programs Northeastern United States of America	Semi-structured interviews Transcendental phenomenological framework	Strong	Some	None reported
**32. Roberts et al. (2011) (2 papers)** Complex Calculations: How Drug Use During Pregnancy Becomes a Barrier to Prenatal Care Women’s perspectives on screening for alcohol and drug use in prenatal care	Women’s perspectives	N = 38 pregnant or postnatal women with current or previous substance abuse	Multiple settings California, United States of America	Semi-structured interviews (n = 20) and focus groups (n = 2) Thematic coding	Adequate	Some	None reported
**33. Schiff et al. (2022) (2 papers)** “You have to take this medication, but then you get punished for taking it”: lack of agency, choice, and fear of medications to treat opioid use disorder across the perinatal period **Work et al (2023)** Prescribed and penalized: The detrimental impact of mandated reporting for prenatal utilization of medication for opioid use disorder	Women’s perspectives	N = 26 mothers receiving treatment for opioid use disorder	A multidisciplinary clinic for perinatal women with substance use disorders Massachusetts, United States of America	Semi-structured qualitative interviews Constant comparative methods	Strong	Some	Some
**34. Stengel (2013)** The risk of being ’too honest’: drug use, stigma and pregnancy.	Women’s perspectives	N = 13 pregnant or postnatal women with substance use disorder, accessing treatment	Community-based maternity care programme British Columbia, Canada	Semi-structured interviews Constructivist Grounded theory	Adequate	None reported	None reported
**35. Stone, R. (2015)** Pregnant women and substance use: fear, stigma, and barriers to care	Women’s perspectives	N = 30 pregnant or postnatal women with substance use disorder	United States of America	In-depth interviews	Strong	Some	None reported
**36. Syvertsen et al. (2021)** Conceptualizing stigma in contexts of pregnancy and opioid misuse: A qualitative study with women and healthcare providers in Ohio	Women and healthcare professionals’ perspectives	N = 28 women of which n = 15 currently pregnant with substance use disorder; N = 18 healthcare professionals	Ohio, United States of America	Semi-structured interviews Life history approach, using Content Analysis	Strong	Some	None reported
**37. Titus-Glover et al. (2024)** The lived experiences of pregnant and parenting women in recovery toward medication treatment for opioid use disorder	Women’s perspectives	N = 11 pregnant and postnatal women	Outpatient clinics at major hospital with specialty clinics, United States of America	Focus groups and individual interviews Grounded Theory Approach	Strong	Some	None reported
**38. Tsantefski al. (2014)** A delicate balance: Intervention with mothers with dual diagnosis and their infants	Women and healthcare professionals’ perspectives	N = 22 postnatal women attending a drug and alcohol service; N = 20 Staff members, of which 2 healthcare professionals, 18 child protection staff	A Women’s Alcohol and Drug Service at a women’s hospital Victoria, Australia	Semi-structured interviews (three timepoints) Content Analysis	Adequate	Some	None reported
**39. Vasilevski et al. (2024)** Barriers and enablers to antenatal care attendance for women referred to social work services in a Victorian regional hospital: A qualitative descriptive study	Women and healthcare professionals’ perspectives	N = 10 pregnant or postnatal women with a referral to social services during pregnancy N = 11 healthcare professionals	Public tertiary maternity hospital, Victoria, Australia	Semi-structured interviews Constructivist Grounded Theory Approach	Strong	Some	None reported
**40. Whittaker et al. (2016)** The burden of care: a focus group study of healthcare practitioners in Scotland talking about parental drug misuse	Healthcare professionals’ perspectives	N = 18 healthcare professionals	Scotland	Focus groups	Adequate	None reported	None reported
**41. Wood (2008)** Taking the baby away. Removing babies at birth for safeguarding and child protection	Healthcare professionals’ perspectives	N = 9 midwives	Acute hospital setting, London, United Kingdom	Semi-structured interviews Thematic based analysis	Adequate	Considered	Considered

* Assessment criteria 1: CASP Checklist

**Assessment criteria 2: Trauma-informed approaches reported in study

*** Assessment criteria 3: Public and patient involvement reported in study

Included studies were published between 1993 and 2024, with study settings predominantly in the Global North, including Cananda (n = 4) [52–55], Germany (n = 1) [56], United States of America (n = 16) [50, 51, 57–74], United Kingdom (n = 7) [9, 49, 75–80] and Australia (n = 12) [13, 15, 81–89]. One study was carried out in Brazil [90]. Nine studies included both women’s and HCPs’ views, twenty-two studies focused on women’s perspectives only and ten studies focused on HCPs’ perspectives only. Most data werecollected through individual interviews and focus groups, representing the views of a total of 1040 women and 307 HCPs. Most studies had a specific vulnerability focus that warranted involvement from CP agencies, of which twenty-four mentioned substance misuse, two homelessness, one intellectual and/or developmental disabilities and one co-occurring intimate partner violence and opioid use disorder [72].

### Quality assessment

Most included studies were categorised as ‘strong’ (n = 30), with eleven studies being considered ‘adequate’. Overall, concerns around methodological quality were mostly situated around a lack of detail regarding the methods of data analysis, clear statement of findings and issues around positionality of the researcher. A similar concern was found when assessing the use of trauma-informed approaches and patient and public involvement in the research design, with only nine studies providing detailed trauma-informed considerations of conducting research with vulnerable women and only five studies reporting on of robust lived experience involvement, either at the stage of study design or sense-checking of study findings. When combining all three assessment criteria, only one study was found to be of strong methodological quality, with robust considerations of trauma-informed approaches and service-user involvement [53]. In contrast, one study was considered adequate with no considerations at all for trauma-informed approaches and service-user involvement, even though its focus was postnatal women with substance use disorders [55].

## Findings

### Phase 1: Data familiarisation and formulating a critique of the existing evidence

During the first phase of familiarisation and iterative coding, we found that similar themes were present in both women’s and HCPs’ accounts, yet only interpreted from one point-of-view. For instance, studies reported on the importance of ‘trust’ for women to feel safe with a HCP and only then would they feel comfortable to disclose the extent of their issues [15, 55, 59, 60, 63, 77, 85]. Conversely, several studies capturing HCPs’ experiences reported that they were fully aware trust was an essential requirement to provide optimal care to women [57, 72, 75, 80–83, 90, 91]. Continuity of care provider was often mentioned by HCPs and women as a pre-requisite to build a relationship of trust and honesty [49, 75, 80–83, 85, 89–91]. However, many studies mentioned judgement and stigma interfering with the process of relationship-building, leaving women distrusting HCPs and undermining disclosure [49, 55, 60, 63, 71, 72, 77, 83, 85]. This ‘catch-22’ of incompatible conditions to facilitate positive interactions was found across several themes and subthemes. Another example is ‘feelings of motherhood’: several studies mentioned how women felt encouraged in their identity as a mother by their HCP and how this empowered them to engage with healthcare and the CP process [15, 53, 57, 60, 63, 67, 68, 75, 92]; Conversely, other studies mentioned women felt their identity as a mother was challenged and scrutinised, leading to distrust and feelings of shame and judgement [51, 55, 60, 71, 72, 75, 76, 83, 85, 87]. As a result, almost all studies discussed the identity of motherhood with a lack of depth and complexity and overlooked the role of healthcare interactions to the cyclical development of feelings of motherhood.

Overall, many of the studies included in our review offered a predominantly static view of healthcare interactions and engagement, and provided a one-dimensional explanation of how these encounters are perceived, both by women and professionals. The complexity and fluctuating appraisals of healthcare interactions over time, based on a myriad of factors both within the individual as well as the wider organisational and societal context, was often not explored in depth. Few studies aimed to untangle how one aspect—for instance fear of detection of substance use—can be a barrier to healthcare at one timepoint, and a facilitating factor at another point, for the same woman, during the same pregnancy [51, 55, 63, 70]. As a result, the strength of this review is to bring these various viewpoints together to create a holistic understanding of how healthcare encounters are constituted, how they fluctuate and what their potential outcomes are.

### Phase 2: Generating a synthesising argument

**Fig 2:**
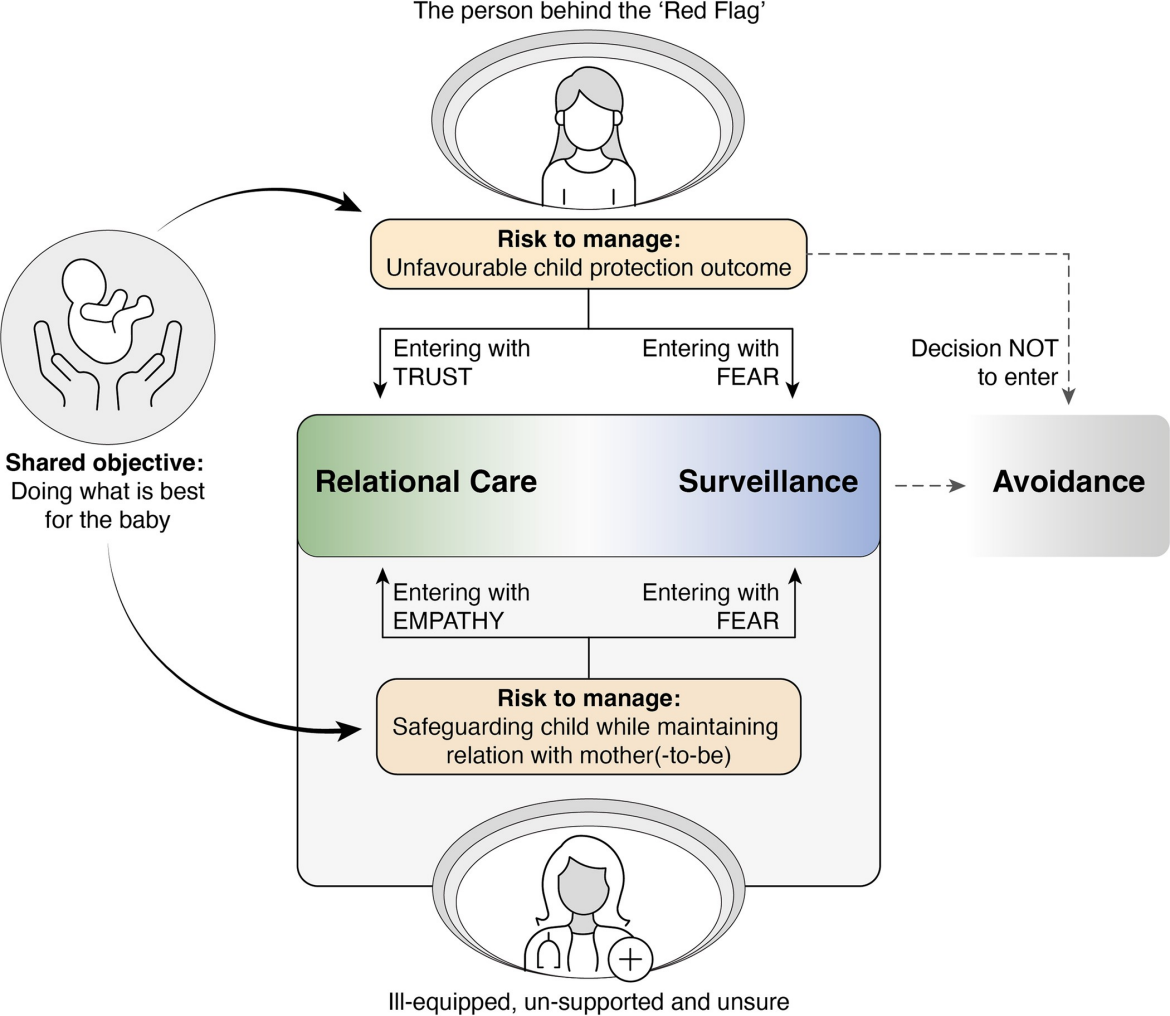
Visual display of review findings.

During the second phase of CIS, we grouped codes together to facilitate the next step of data synthesis, i.e. creating ‘synthesising theoretical constructs’. We identified patterns of interactions between women with CP involvement and HCPs involved in their care, and grouped individual, interpersonal, organisational and societal conditions that influenced these reciprocal relations, either negatively or positively. Our review findings are visually displayed in Fig 2 and represent the key synthesising constructs that were distilled from our interpretation of included studies. Key concepts are illustrated with representative quotations from included studies in the following paragraphs.

### ‘Doing what is best for the baby’–One objective, two sides of the same coin

We identified from the majority of included studies that both women and HCPs had one common objective: to do what is best for the (unborn) baby [15, 54, 55, 60, 62–64, 66–68, 70, 73, 76–78, 80–82, 85–87, 89].

*"We are here because we want to do the right thing. That is the right thing by ourselves and the right thing by our babies. It’s like a business partnership, a two-way street. You each want the best outcome for the child.” (Pregnant woman with substance addiction, Australia) [85]*

*“I just want the best for him. … I don’t want him to be exposed to that life. I don’t want him to think I neglected him at all, because I didn’t. I changed my whole life around for him, and myself, but him first.” (Postnatal woman with substance use disorder, USA) [51]*

*"What I feel my role is and that is to protect the child at all costs really." (Midwife, UK) [79]*

However, both women’s and HCPs’ actions were underpinned by juxtaposing risks they needed to manage. The dichotomous interpretation of how to achieve the common objective acted as two sides of a coin: For women, managing the risk of an unfavourable outcome by child protection services was going to determine if they felt able to safely enter the healthcare system, or whether it was preferrable to avoid contact with healthcare professionals [15, 51, 55, 60, 62–64, 66–68, 70, 72, 76, 77, 85–87].

*"Knowing that they were gonna test me for drugs, that’s what scared me…That’s why I didn’t go to prenatal care…I didn’t want to lose my baby." (Pregnant heroin user, USA) [62]*

*“There are women who don’t seek care because they don’t want [Child Protection Agencies] involved. Same is true with intimate partner violence, they’re ashamed, they don’t want [Child Protection Agencies] involved. So again, that being co-occurring, I think the biggest barrier.” (Healthcare professional, USA) [72]*

Several studies reported that women’s determination to ensure the baby’s wellbeing acted as a motivator to enter healthcare, even when they feared subsequent CP involvement [15, 55, 64, 66, 67, 73, 85, 89].

*"[Fear of Child Protection Services] made me not want to go, but because I was high risk, there was a greater chance […]. I care more about my son being ok…so, I never missed a[n] appointment." (Woman with substance addiction, USA) [67]*

Conversely, for HCPs, the dilemma was of a different nature, and the challenge of managing the risk of harm to the baby while also maintaining a relationship with the mother(to-be) was repeatedly mentioned [54, 72, 78, 79, 81, 82]:

*"I have a very important role with regards to the women, but I am also very in tune with the fact that I have a professional duty to protect the baby and 9 times out of 10 babies are not removed unless there is a good reason to do it and I have to keep that there to be able to do my job." (Midwife, UK) [79]*

*"Even when I know that it is for the best reason, it’s still hard as there is still a woman with dreams." (Midwife, Australia) [81]*

In some situations, the all-encompassing professional focus on the wellbeing of the foetus or baby, with less regard for the health concerns and needs of the mother(to-be), highlighted the dehumanisation of pregnant people, especially when using illicit substances [50, 74]:

*“I think it might just be being pregnant in general but you don’t always get talked to. You’re almost talked as if you are a baby maker, like baby machine […]. It’s like, they almost like talk and look at your stomach, like when they are talking to you. It’s just like, you lose that sort of normal talking thing.” (Woman with substance use disorder, USA) [74]*

### Ill-equipped, un-supported and unsure

We found an array of factors that impacted HCPs’ behaviours, attitudes and expectations when caring for women with CP involvement. Their assessment and strategy on how to enter and maintain this relationship was based on their level of confidence and expertise and personal beliefs and biases [54, 57, 72, 78].

*"I feel like [alcohol], it’s so harmful, right? Those babies that come out addicted to cocaine, methadone, whatever, they end up doing okay. The FAS [fetal alcohol syndrome] babies don’t do okay. I struggle with the moms that have been drinking throughout their pregnancy." (HCP, Canada) [54]*

A critical requirement for delivering high-quality, evidence-based and personalised care, was access to robust supervision and support [75, 78, 79], especially in case of an unexpected poor outcome [78].

*“Whenever some child dies, they go round looking for somebody to hang out to dry and introduce another set of tick boxes and it doesn’t change anything meaningfully." (General Practitioner, Scotland) [78]*

Equally important was access to relevant training so HCPs felt equipped to provide high standard care in the context of safeguarding. HCPs reported this aspect of clinical practice was often lacking in their pre- and post-registration training and they were often asked to practice outside the scope of their professional role [49, 57, 59, 71, 72, 78, 92].

*“There’s loads of specialist people that deal with people with learning disabilities, help me, please tell me where I’m supposed to go with it, ’cause I didn’t know… I tried, loads of times… But it just was falling on deaf ears… So it was really frustrating." (Midwife, UK) [75]*

*“I like to stay in my own lane. I can only talk to them [patients] about the recovery aspect, and then I point them to social work. As soon as they say DV [domestic violence], I’m like, “let’s bring the social worker in.” (Healthcare provider, USA) [72]*

This was echoed by women, who pointed out the importance of adequate training of HCPs in order to receive personalised and compassionate care from them:

*“I find a lot of them [nurses], you know, are not very sympathetic, they don’t want to help you…I’m finding they need to train their staff more to be more understanding of us not just as parents and mothers, but as human beings, you know, how to interact with us with any situation and be more helpful when we’re asking questions instead of judging us or looking at us weird." (Postnatal woman, Canada) [52]*

Overall, the task of providing high quality healthcare while safeguarding both mother and baby was described as daunting, with little recognition by the wider organisation or healthcare system of the ‘burden of care’ this constituted [9, 72, 78, 79, 81, 82].

*"None of us have got adequate resource to do the job we’re being asked to do, to provide adequate child protection and parenting support. I certainly don’t in a 10-minute appointment, when I’ve got to get a methadone script done at the same time." (General Practitioner, Scotland) [78]*

*“We’re under resourced with this [current policy]. In order to make something work, the state would have to put in enough money in order to actually support it." (HCP, USA) [61]*

### The person behind the ‘Red Flag’

Similar to HCPs, women’s considerations whether or not to access healthcare during pregnancy and the postnatal period depended on a range of factors that would influence their decision-making. Many women had previous adverse life experiences, including past experiences with CP agencies. Previous contact with CP agencies was in most cases documented as a ‘red flag’ on their medical record and immediately shaped a perception of them as a risk and trouble-maker, which in response made them feel apprehensive of healthcare and healthcare professionals, with little confidence in a positive outcome [50, 51, 53, 56, 58, 62, 63, 71, 78, 83, 84, 86, 89, 92].

*"I was an instant red flag at any hospital in [large city]. It wasn’t even a matter of, ’Hey, prove yourself. Show us that you can do it,’ until after they apprehended her." (Woman with learning disabilities, Canada) [53]*

*“I didn’t know [what my reaction was]. I was more scared because I didn’t want [Child Protection Services] coming to take …the baby. I didn’t know if they were going to let me keep the baby or if they were going to take custody of the baby like they did with the other [kids]…” (Woman with substance use disorder, USA) [51]*

In several studies, HCPs seemed aware that women with CP involvement often had experienced traumatic and difficult upbringings, and blame was not appropriate to foster positive healthcare relationships [61, 71, 81, 83, 92].

*"A lot of our clients have, maybe, been brought up in care, been in very abusive relationships as a child and they didn’t actually have any idea of what was expected of a ’normal’ parent, they don’t know what mums and dads are meant to do." (Health Visitor, Scotland) [78]*

*"Their background exposes them to situations where they haven’t been able to make the right choices and places the baby at risk. But by separating another child from its family aren’t we are just creating an ongoing cycle of generational trauma?" (Midwife, Australia) [92]*

Women wanted to be seen as more than a ‘red flag’, without fear or shame, but for who they truly were, with all their strengths and flaws. Instead, they had to navigate challenging power dynamics [15, 50, 52, 58, 61, 63, 69–71, 84, 85, 92].

*My personal information, which the doctor had in front of her, included my educational details [Master of Science degree] and employment history, yet that didn’t appear to come into it. I could have talked with her on her own level but in her eyes I was just another pregnant woman with a problem." (Pregnant woman with drug addiction, Australia) [85]*

*"Even though I’m prescribed the medication and I’ve been sober and I’ve been doing everything right, it doesn’t matter. When I get [to the hospital] they can do whatever the hell they want. And I can’t say a damn thing about it because I’m a drug addict and I will always be a drug addict and no matter if I’m on Suboxone or not, to them it’s still a drug." (Pregnant woman with substance use disorder, USA) [71]*

Shame and stigma were often internalised and would prevent women from fully disclosing how they were feeling, what they were going through or how they viewed themselves, including feelings of self-judgement and guilt [15, 51, 53, 55, 57, 63, 85, 92]. This in turn would affect whether they would enter the healthcare system and if so, how confident or fearful they were when interacting with HCPs.

*"I never told the doctors that I had a disability. [I] felt ashamed. Felt embarrassed. Didn’t want to admit to myself -well, I knew but I was still trying to hide it from myself too, at the same time, and I didn’t want people looking at me different because I had a disability." (Woman with intellectual disability, Canada) [53]*

### A fractured multi-agency system: navigating the maze

Healthcare interactions and CP processes take place in multi-agency systems. Unfortunately, many studies reported that navigating this complex maze was challenging for both women and HCPs, without oversight of agencies involved or clear communication between them [9, 54, 72, 75, 79, 81, 82, 88, 89, 91].

*"I felt like I was banging my head up against a brick wall, to try and find the help and support, the appropriate help and support for her." (Midwife, UK) [75]*

*"We’d been waiting for ages for this placement […] as soon as we got out it was like, oh my God, they (Child Protection Practitioners) haven’t even done anything yet. They weren’t communicating with us at all." (Postnatal woman, Australia) [88]*

Both women and HCPs reported they were faced with unrealistic expectations, that set them up to fail. Several studies explicitly mentioned how women found attending a vast number of appointments particularly challenging, especially when they were already feeling vulnerable or overwhelmed [73, 76, 77, 93].

*“I’ve gotta now go to the [methadone dispensation] clinic every single day with my baby and hope that whatever this person’s coughin’ to the left and to the right of me isn’t somethin’ like pneumonia. Why is it that I just had a baby three days ago and they were able to dispense me my medication in the hospital, but yet I come home from the hospital and you expect me to waddle my open body with my child in this car seat and carry it, no doubt?" (Postnatal woman with opioid use disorder, USA) [93]*

HCPs echoed how they had witnessed the pressure on women to meet unrealistic expectations, with no guarantee on a positive outcome.

*"The woman herself, she jumped through hoops during the pregnancy. Went to three or four different courses, the Triple P (Positive Parenting Program) and had some counselling. She worked with Community Services and she had a case worker. They still took the baby into care." (Midwife, Australia) [81]*

Navigating these positions of power was further compromised by the impact of colonialism and racism and both women and HCPs reported how discrimination based on race, ethnicity and cultural background was perpetuated within the healthcare system [52, 53, 61, 63, 69, 71, 74, 83, 86, 92].

*"My last piss test was positive for meth… So they tell me they want to support me with my drug use, but hell no, they are removing my baby from me…It is because I am Aboriginal you know…" (Pregnant woman attending a Drug and Alcohol service, Australia) [86]*

*“There’s so much stigma around drug use and I think it’s very situational and then there’s a lot of institutional racism that’s based around drug use. So, I think a doctor or healthcare provider in general is going to look at someone who is not white and more readily do a drug screen on them than someone who is white to do a drug screen. I think there is so many institutional factors regarding our society that it’s gonna bias people towards testing and reporting, and I think it’s extremely unfair. I think there’s a lot of things that we have to change from an institutional perspective to actually be fair to patients." (HCP, USA) [61]*

Our interpretation of included studies resulted in a theoretical framework of three types of healthcare interaction for women with CP involvement: Relational care, Surveillance and Avoidance. In previous paragraphs we described the factors that would determine whether HCPs approached women with kindness, respect and compassion [9, 15, 54, 57, 60, 67, 68, 81, 82, 85, 89, 90] or with fear, stigma and judgement [9, 49, 51, 53, 55, 57–59, 61, 63, 66, 71, 76, 85]. Women knowingly entered the healthcare system, aware of the risk this might pose towards their parental rights yet motivated by the belief that engaging with healthcare professionals and compliance with hospital protocols (such as urine drug screening) would put them in the best stead to manage this risk [59, 60, 63, 65, 70, 78].

### Relational care–Together, I can do this

Where women and HCPs engaged with trust and compassion, interactions were positively appraised, and ‘Relational Care’, our first type of healthcare interaction—would be facilitated.

*“Weird, and like at first, I thought I was crazy. I thought something was wrong because I wasn’t used to it… . People being nice to me about it. Not even just being nice, like, "Oh, good for you." It’s like people just treating me just like a person, not like a drug addict, not a loser. It was really uncomfortable in the beginning, which is weird… . I just felt like it wasn’t me. I don’t know how to explain it, but like they were treating me like I wasn’t used to being treated, so I felt like I wasn’t myself, but I got used to it and I think I started to feel like, Okay. I deserve to be treated like a person. I deserve to be treated, you know, because I am doing the right thing. And it’s hard, but it’s good… . " (Postnatal woman using opiates or illicit substances, USA) [60]*

As women gained trust in their HCPs, they became more forthcoming with disclosing the issues they were facing, and more receptive of support void of judgement and punishment. [15, 53, 54, 56, 57, 60, 63, 73, 74, 80–82, 85–87, 89, 92].

*"She [midwife] was awesome. She turned off every computer in her office, she didn’t write anything down, she was like, ’Tell me anything you want to tell me, even if it’s about Children Aid, just let it loose,’ you know? And I was able to talk to her and I didn’t feel like she was writing anything down at the time. So I didn’t have to stress that, ’Oh, no! What is she writing?’ kind of thing. So it kind of made me feel more at ease, to know that she was actually sitting there, listening to me." (Postnatal woman, Canada) [52]*

Continuity of care provider was mentioned in several studies as a crucial element to build this reciprocal relationship of trust [49, 72, 75, 80–83, 85, 89–91].

*“I get peace of mind having my appointments so close together, and I only see the one midwife, so I’m not explaining everything over and over again.” (Woman, referred to Social Work Services in pregnancy, Australia) [89]*

*“What works is the ability to meet them [patients] early in the pregnancy before they start doing more regular visits so that you’ve established a rapport. In order to provide I think adequate support for these women, is for them to trust you and get that rapport going which means giving them time in an area when they’re on their own, so as I said before, not with the partner or the children" (Midwife, Australia) [83]*

Conversely, lack of continuity of carer and fragmented care was seen as an obstacle to achieve the consistency required to underpin any relationship of trust with HCPs.

*"We just got thrown everywhere, alcohol and drug services, different midwives, doctors and social workers. Seeing somebody different every time you went made it harder and confusing. We had to keep telling our story over and over, so that was quite frustrating, as was dealing with the different people’s attitudes." (Postnatal woman, Australia) [92]*

The impact of a trusting relationship with HCPs and compassionate and respectful care was profound, and instilled or reinforced a belief in oneself as a ‘mother’, which many women had not experienced before [15, 53, 57, 60, 63, 67, 68, 73, 75, 92].

*"I think she thinks I’m a great mother, and a strong woman for doing what I’ve done. Partly because she has told me that I’m doing great, staying in the program. But, she just seems proud, you know what I mean?… Of what I was when I came in, and what I have accomplished now." (Postnatal woman, USA) [60]*

*"I saw her [Child and Family Health Nurse] for years… she got it into my head, you’re a mum; it’s no different being on methadone. She dealt more with my problems than my baby’s and saying, ’You’re a mother, you’re doing a great job’. It’s just that reinforcement, it can just be those words that really help you.” (Postnatal woman under care of opioid treatment clinic, Australia) [15]*

### Surveillance–box-ticking and dip-sticking

In contrast, where women entered healthcare with fear of potential repercussions, this was often mirrored by professionals’ fear. In these circumstances stigma and judgement could find fertile soil.

*"The women come in waiting to be judged. They assume from the get-go that we are judging them for their addiction… I think they are a little shy, or even afraid of, interacting with health care professionals because of the fear of judgment." (Woman with substance use disorder, USA) [71]*

Consequently, interactions were negatively appraised and healthcare encounters were reduced to ‘surveillance’ [15, 49, 52, 55, 56, 60, 62, 65, 69, 77, 78, 85, 86]. This was characterised by a reciprocal pattern of ‘compliance’, whereby both women [55, 57, 63, 65, 67–69, 71, 76, 77, 85, 88, 93] and HCPs [9, 57, 75, 79, 81, 82, 92] felt forced to follow outlined processes, resulting in a lack of shared decision-making and opportunity to disclose concerns [55, 78].

*"This week I’m supposed to provide three clean urines and they’ll leave me alone but I haven’t […] so I will be lying to them and telling them I have to go somewhere. I know at the moment, the way I am, if Human Services did come today, and I haven’t done my urines, he will be taken off me […] I need to go into detox but I don’t want to tell them because I don’t want them to take him away […] No, I don’t trust them." (Postnatal woman attending Drug and Alcohol Service, Australia) [88]*

*"We do an awful lot of box ticking… . but actually how much impact is that having on the outcome of that child, is it still hell on wheels when the child goes home? (Health Visitor, Scotland) [78]*

Women were facing unrealistic expectations [63, 69, 76–78, 81, 82, 85, 91], and lack of clear communication and clarity about processes led to further relationship breakdowns. Several studies reported how women felt they were treated differently compared to other women, and as a result felt their identify as a ‘mother’ was challenged or even denied [15, 55, 57, 59, 60, 69, 71, 83, 85, 87].

*You just knew that you were not going to be treated equally and like other pregnant women and accepted for who you were. I mean after all I was at the clinic because of drug problems, and that was never forgotten [by the staff]." (Pregnant woman, Australia) [85]*

*"Just because I had her on methadone, like I must be a bad mother, and like don’t take any notice of me; like I don’t want the best for my child, how could I possibly love her on methadone?!" (Woman under care of an opioid treatment clinic, Australia) [15]*

HCPs equally felt pressure to comply with system expectations and felt at times deeply uncomfortable with their involvement in CP processes. This was particularly the case when they were asked to step outside their scope of practice, when boundaries around professional roles were blurred or when HCPs felt complicit in secrecy around the pending removal [13, 72, 75, 78, 79, 81, 82].

*"I was told by the social worker not to let [name of patient] know that they were planning to remove her baby that morning, I didn’t like being involved in that at all. I did not disagree with the reasoning as to why the baby was being removed, just the lying about it to the woman. I told them I would play no part in it but in the end I did, because I had no choice." (Midwife, UK) [9]*

*"And I think that once the baby is born, I feel strongly, I feel that it is not my role to tell the client about her baby not being with her, I don’t think that’s my role, but I don’t have any qualms in being the one to have to take the baby from her, as long as she knows beforehand that this is what’s happening." (Midwife, UK) [79]*

HCPs reported being deeply uncomfortable when ‘committing’ this ‘professional betrayal’, i.e. when they were knowingly untransparent or deceitful about their actions, to the detriment of the CP outcome for the women in their care.

*“I get it but still it’s very tough. You feel really deceptive not telling them… it feels wrong, I’m lying and not being truthful. My role is to advocate for the woman and when I’m withholding information it goes against my clinical practise. Concealing it gives the woman a false sense of security and immediately you have a breakdown in trust with them." (Midwife, Australia) [92]*

Women were left devastated and disillusioned and, in some cases, disengaged with healthcare as a result [15, 55, 60, 62, 63, 70, 87].

### Avoidance–Staying on top by going under the radar

While some women disengaged with healthcare after experiencing ‘professional betrayal’, others avoided healthcare altogether from the start, as it did not feel safe to enter these healthcare relationships in the first place. Both situations led to our third type of healthcare interaction, i.e ‘Avoidance’, through which women managed the risk of an unfavourable CP outcome by steering away from HCPs and the healthcare system as a whole [15, 51, 55, 60, 62, 63, 70, 87].

*"If you have another [drug-exposed] child within a three-year period, even if you’re staying clean and sober, your child will be taken from you, and can be automatically be placed for adoption…[it is a] state policy. I wanted to come here [to the treatment program] and there wasn’t an opening… I didn’t go to my doctor at that time [in pregnancy] because of my name being on that list…I was really scared of that…that’s what kept me from going to prenatal care." (Pregnant woman in residential treatment for alcohol or drugs dependency, USA) [62]*

*"You don’t want to sit there and say I’m a drug addict, I’m about to have a baby please take it off me. You know, that’s your fear. So, I didn’t access anything (antenatal and treatment) at all. I didn’t seek any medical assistance until the seventh month, out of fear" (Woman with substance use during pregnancy, Australia) [87]*

## Discussion

Our review findings provide an in-depth analysis of the experiences of both women and HCPs when seeking or providing healthcare in the perinatal period, while facing CP involvement. We found that both women and HCPs have a common overarching objective at the start of healthcare interactions, i.e. to do what is best for the baby, with two juxtaposing risks to manage for either women or HCPs. We identified three types of healthcare interactions, Relational care, Surveillance and Avoidance, each characterised by a range of factors. It is important to note that the appraisal of healthcare interactions is not static and can easily fluctuate based on the quality of individual encounters and whether trust is upheld. Furthermore, when women and HCPs engage with one-another, healthcare interactions are often not dichotomously reduced to ‘Relational care’ or ‘Surveillance’. Even in the most positively appraised healthcare interactions, there remain elements of surveillance and compliance, but they are accepted by women as ‘part of the package’.

Another key finding is that healthcare interactions do not exist in a vacuum, but are determined by a constellation of factors on an individual, inter-personal, organisational, and societal level. We identified various factors on these different levels, such as HCPs’ access to training and supervision, feelings of self-worth and confidence, previous experiences and trauma, etc. and their complex interaction will determine women and HCP’s starting positions when engaging with one-another. In addition, these factors will continue to impact an ongoing relationship throughout pregnancy and the postnatal period.

Our findings are novel, as they reveal the complexity and multi-facetted reciprocal nature of healthcare interactions during the perinatal period in the context of child protection. Based on our review findings, we identified four specific areas for improvement that are essential to achieve relational care in every perinatal healthcare interaction when facing CP involvement.

Firstly, our findings underscore the need for building a workforce across the perinatal healthcare landscape that is confident in safeguarding while upholding principles of trauma-informed care. Our review highlighted that women with CP involvement remain on the receiving end of stigmatising attitudes by HCPs. Misconceptions around vulnerabilities such as addiction, disabilities, homelessness etc. can compound stigma that many women in these circumstances already face and often disregard the level of trauma they have experienced. Trauma-informed approaches in healthcare move away from such punitive attitudes but acknowledge previous trauma and adversity and their enduring consequences [94]. As a result, where women are approached with respect and compassion and their previous life adversity is acknowledged, they feel seen and heard, which can instil strength, motivation and a sense of deserving motherhood. Unfortunately, pre- and post-registration training in safeguarding and relevant CP processes is inconsistent yet essential to ensure HCPs feel confident and adequately equipped to carry out this challenging part of their clinical practice. Recent guidelines such as Mason et al. (2023)’s ‘Best practice guidelines for when the states intervenes at birth’ [95] and the ‘Birth Charter for women with involvement from Children’s Social Care’ by Birth Companions [96] have specified care for women with CP involvement should be holistic, culturally appropriate, trauma-informed, trauma-responsive and equitable [95, 96]. Our findings have identified a similar required skillset to provide compassionate relational care, concordant with these guidelines. As such, training opportunities for HCPs should reflect the range of skills to ensure safeguarding is conducted in a responsive and meaningful way, meeting both the needs of the child and its mother.

Secondly, our review found that women of Black or Indigenous backgrounds reported systemic racism when accessing healthcare during the perinatal period, and that they believed racist attitudes and beliefs from their HCPs negatively impacted their chances of a positive CP outcome. Racial and ethnic disparities are prevalent in both (maternity) health care and CP referrals and require an intersectional approach to be fully understood [97–99]. Across the world, women of Indigenous and racialised groups experience poorer (reproductive) health [100] and have disproportionately more contact with CP services, with higher rates of child removal [99, 101, 102]. Calls to acknowledge the intergenerational trauma of child removal in indigenous communities in settler countries (e.g. Canada, USA, Australia, New Zealand etc) and the role of HCPs in perpetuating disparities in maternal health outcomes and social care referrals should be urgently addressed in an effort to tackle these persistent inequalities and improve healthcare for these communities [103, 104].

Thirdly, our findings laid bare the complexities and fragmentation of multi-agency working for women accessing healthcare and facing CP involvement. Poor information sharing between multi-agency partnerships has been identified as a compounding factor that can lead to serious harm, abuse or death of a child [105]. In addition, it has also been described as a particular challenge in the perinatal period by health and social care professionals [8, 11, 12]. Post-COVID strain across health and social care services, including maternity and mental health services, in combination with increased regulatory and public scrutiny have compounded defensive safeguarding practice. In England, public funding for safeguarding and children’s social care has been static since 2009–2010, while numbers of children in foster care and those with social services involvement have significantly risen [106]. In these challenging times, marked by increased childhood poverty [107], and reduced access to Early Years support and prevention services, families with social risk factors are more than ever reliant on universal services that truly work together to support them. Information-sharing and multi-agency collaboration must be timely, secure, relevant and proportionate, not just for families at the heart of it, but for every professional involved in their care.

Finally, our analysis highlighted the need for transparency and clear information throughout the entire perinatal healthcare journey, when CP agencies are involved. Pregnant women need to be informed about what to expect in light of a referral to CP agencies, what possible outcomes a referral might have and what the subsequent steps of the process might be. Such conversations can be difficult to have, especially in the face of fractured, strained and/or under-resourced services, lack of professional safeguarding training and expertise and fear of endangering the relationship with women in their care. However, our findings highlighted that transparency has the potential to alleviate some of the professional burden as well as provide a clear framework of expectations for women who face CP involvement.

## Strengths and limitations

This qualitative evidence synthesis is the first to provide a holistic overview of the experiences of healthcare in the perinatal period when CP processes are at play. The synthesis includes a wide range of views from both women as well as HCPs from various healthcare settings that provide care in this context. Our search strategy, with its broad search terms and inclusive approach, facilitated the identification of a wide range of highly relevant studies for inclusion. Our rigorous two-phase approach to analysis of extracted data provided a novel interpretation of the existing qualitative evidence. The findings were strengthened through sense-checking by the advisory panel of women with lived experience of infant removal, and explicit positionality, reflexivity, multi-disciplinary review team composition and continuous team discussions.

However, there are limitations to this review. Search results did not retrieve any relevant studies from low- or middle-income countries. While many of these countries have less resources to ordain, implement or enforce CP legislation, it does not mean these processes are entirely absent in such countries. Nevertheless, our review was unable to examine women’s experiences of perinatal health care in low- or middle-income countries when facing child protection.

## Conclusions

This review provides insight into the complex and reciprocal nature of healthcare interactions during the perinatal period when facing CP processes. These interactions do not occur in a vacuum and are influenced and impacted by a myriad of factors at the individual, inter-personal, organisational and societal level. As such, the intersection of these factors will affect whether healthcare interactions are appraised positively or negatively. Through our review findings, we have identified four important areas for care improvement for pregnant or postnatal women with CP agency involvement. Building a trauma-informed workforce that is confident in safeguarding is essential to facilitate positive perinatal healthcare experiences. Looking at the wider healthcare system, the systemic impact of post-colonialism and racism, has been acknowledged as a priority to address existing reproductive health inequalities of Indigenous and racialised groups [100, 104, 108]. Our review has highlighted this should also be extended to safeguarding practice and processes of CP when accessing healthcare. In addition, there is a need to strengthen multi-agency working so HCPs feel secure and supported in this challenging aspect of their professional role. Finally, trust and transparency are key to facilitate relational care, which can improve healthcare experiences and engagement, alongside patient and healthcare professionals’ satisfaction. A clear framework of mutual expectations can play a role to facilitate a continued relationship between service user and provider based on trust, transparency and accountability and advance equity in perinatal healthcare.

## Supporting information

S1 Table PRISMA 2020 checklist. (DOCX)

S2 Table Search strategy. (DOCX)

S3 Table NVivo codebook. (DOCX)
