# Correction: Healthcare experiences of pregnant and postnatal women and healthcare professionals when facing child protection in the perinatal period: A systematic review and Critical Interpretative Synthesis

**DOI:** 10.1371/journal.pone.0357012

**Published:** 2026-08-25

**Authors:** Kaat De Backer, Hannah Rayment-Jones, Billie Lever Taylor, Tamsin Bicknell-Morel, Elsa Montgomery, Jane Sandall, Abigail Easter

The first author’s initials appear incorrectly in the citation. The correct citation is: De Backer K, Rayment-Jones H, Lever Taylor B, Bicknell-Morel T, Montgomery E, Sandall J, et al. (2024) Healthcare experiences of pregnant and postnatal women and healthcare professionals when facing child protection in the perinatal period: A systematic review and Critical Interpretative Synthesis. PLoS ONE 19(7): e0305738. https://doi.org/10.1371/journal.pone.0305738.
