# Supplementary material for: Healthcare experiences of pregnant and postnatal women and healthcare professionals when facing child protection in the perinatal period: A systematic review and Critical Interpretative Synthesis
Source: PLoS One. 2024 Jul 3;19(7):e0305738. doi: 10.1371/journal.pone.0305738 (PMC11221698; doi:10.1371/journal.pone.0305738)
Supplement: S2 Table — (DOCX) [file pone.0305738.s002.docx]

**S2 Table. Search strategy, in line with research question based on PerSPE©TIF Framework (Noyes, Booth et al.2009)**

| **No.** | **Framework** | **Free text terms** | **MeSH terms** |
| --- | --- | --- | --- |
| 1 | **Perspective:**  Pregnant and postnatal women, healthcare professionals | mother* OR wom?n OR pregnan* OR parent* | **Medline & Embase**: exp mothers/ or exp pregnant women/  **CINAHL: (**MM ”Expectant Mothers”) or (MM “Mothers”) or |
| 2 | **Setting:**  Healthcare settings | Not included as separate concept as captured by phenomenon of interest |  |
| 3 | **Phenomenon of interest**:  Healthcare | service* OR support OR practice OR intervention* OR program* OR treat* OR therap* OR care OR counsel* | **Medline:** exp Patient Care/  **Embase:** exp perinatal care/ or exp outpatient care/ or exp prenatal care/ or exp postnatal care/ or exp universal health care/ or exp hospital care/ or exp primary health care/ or exp mental health care/ or exp maternal care/ or exp holistic care/ or exp emergency care/ or exp patient care/ or exp medical care/ or exp intrapartum care/ or exp community care/ or exp nursing care/  **PsychInfo**: exp Managed Care/ or exp Patient Centered Care/ or exp Health Care Access/ or exp Health Care Delivery/ or exp Health Care Policy/ or exp Health Care Utilization/ or exp Health Care Seeking Behavior/ or exp Long Term Care/ or exp Primary Health Care/ or exp Trauma-Informed Care/ or exp Health Care Services/ or exp Intensive Care/ or exp Prenatal Care/ or exp "Quality of Care"/ or exp "Continuum of Care"/  **CINAHL:** MM “patient care+” |
| 4 | **Environment:**  Ongoing Child protection processes | “child remov*” OR “infant remov*” OR child welfare OR infant welfare OR “child ADJ3 custody” OR “child ADJ3 safeguard*” OR child protect* OR care proceeding* OR “loss ADJ3 custody” OR “custody ADJ3 remov*” OR “remov* ADJ2 birth” OR “loss ADJ1 parent* right*” OR assumption of care OR “separation ADJ2 birth” OR tak* into care | **Medline & Embase:** exp child protective services/ or exp child abuse/ or exp child welfare or exp infant welfare/  **PsychInfo:** exp Child Abuse/ OR exp Child Custody/ OR exp Child Welfare/ OR exp Child Neglect/ or Protective Services/ or Social Services/  **CINAHL:** MH "Child Welfare+"/ OR MH "Child Abuse+" or (MM “Child Custody”) |
| 5 | **Timeframe:**  Perinatal period (pregnancy up to one year after birth) | antenat* OR prenat* OR pregnan* OR postnat* OR perinat* OR postpart* OR peripart* OR birth | **Medline:** exp pregnancy/ or exp parturition/ or exp postnatal care/ or exp perinatal care  **Embase:** exp pregnancy/ or exp prenatal period/ or exp perinatal period/ or birth/  **PsychInfo:** exp Pregnancy/ or exp Postnatal Period/ or exp Perinatal Period/  **CINAHL:** (MM "Prenatal Care") OR (MH "Postnatal Care") OR (MH "Postnatal Period") OR (MH "Pregnancy+") OR (MH "Sexual Reproduction Periods+") |
| 6 | **Findings:**  Qualitative evidence | qualitative* OR interview* OR thematic* OR ethnograph* OR "grounded theory" OR phenomenolog* OR experiential OR "focus group*" OR discourse* OR "conversation analysis" OR "framework analysis" OR narrative* OR "lived experience*" OR account* OR hermeneutic OR "mixed method*" OR "mixed-method*" OR "semi-structure*" OR "semi structure" OR IPA OR interpretative* OR "content analysis" OR "guided discussion*" OR "group discussion*" OR "open-ended" OR "open ended" OR "audio-record*" OR "audio record*" | **Medline:** exp qualitative research/  **Embase:** exp qualitative research/ or exp qualitative analysis/  **PsycInfo:** exp Qualitative Methods/  **CINAHL:** MH "Qualitative Studies+" |
