# Supplementary material for: Healthcare experiences of pregnant and postnatal women and healthcare professionals when facing child protection in the perinatal period: A systematic review and Critical Interpretative Synthesis
Source: PLoS One. 2024 Jul 3;19(7):e0305738. doi: 10.1371/journal.pone.0305738 (PMC11221698; doi:10.1371/journal.pone.0305738)
Supplement: S3 Table — (DOCX) [file pone.0305738.s003.docx]

# S3 Table. NVivo Codebook

| Key themes or concepts | Sub-themes |
| --- | --- |
| A fractured multi-agency system | Blurry professional boundaries  Lack of clarity (communication, process and consequences)  Managing (unreal or unclear) expectations  Negotiating positions of power  Perpetuating colonialism and racism  Professional compliance - Just following the rules  Wrap-around care |
| Acceptance of care | Motivations for care-seeking |
| Avoidance of healthcare | Excluded from care  Looking for support, receiving none  Peer-support  Postnatal vacuum of support |
| Barriers to engage or accept care | Access (physical and practical) to care |
| Shared motivation - Doing what is best for baby | Being a good mother (incl. breastfeeding)  Breastfeeding  Concerned about baby's health and wellbeing |
| Factors impacting HCPs attitudes | Access to supervision and support  Advocacy and empowerment  Burden of care  Confidence, knowledge and expertise  Going the extra mile (HCPs)  Training needs |
| Factors impacting women's attitudes | Coping strategies  Partner issues (incl. DA)  Previous adverse life experiences, incl. with Child Protection Agencies  Adverse life experiences  Self-appraisal  Guilt and self-blame  Inner strength and worth |
| Managing risk | Fear of custody loss  Fear of detection |
| Relational care | Flexibility and adapting care  Honesty and disclosure  Kindness, respect and compassion  Person-centredness  Reinforcing motherhood - been seen as a mother  Speaking up, feeling empowered  Shared decision-making  Support free from judgement and punishment  Trauma-informed care  Trust as an essential requirement  Value of Continuity of care provider |
| Surveillance | Being treated 'other', not as a mother  Labelled as 'at risk', too complex  Seen as hopelessly chaotic  Pragmatism and compliance  Medication compliance  Professional betrayal  Stigma and judgement |
